# Comprehensive, Genome-Wide Identification and Expression Analyses of Phenylalanine Ammonia-Lyase Family under Abiotic Stresses in *Brassica oleracea*

**DOI:** 10.3390/ijms251910276

**Published:** 2024-09-24

**Authors:** Umer Karamat, Juxian Guo, Shizheng Jiang, Imran Khan, Mengting Lu, Mei Fu, Guihua Li

**Affiliations:** Guangdong Key Laboratory for New Technology Research of Vegetables, Vegetable Research Institute, Guangdong Academy of Agricultural Sciences, Guangzhou 510640, China; umerkaramat23@gmail.com (U.K.); guojuxian@gdaas.cn (J.G.); jiangshizheng2021@163.com (S.J.); dh18006@yzu.edu.cn (I.K.); lumengtting@163.com (M.L.)

**Keywords:** phenylalanine ammonia-lyase, PAL, *B. oleracea*, cold, abscisic acid, abiotic stress

## Abstract

Phenylalanine ammonia-lyase (PAL) acts as the rate-limiting enzyme for anthocyanin biosynthesis through the phenylpropanoid pathway, a crucial component of plant secondary metabolism. The *PAL* gene family plays a crucial role in plants’ defense and stress responses, but its in silico identification and expression analyses in *Brassica oleracea* under different abiotic stresses remain unexplored. In this study, nine *BolPAL*, seven *BrPAL*, four *AtPAL*, and seventeen *BnPAL* genes were obtained from the genomes of *B. oleracea*, *Brassica rapa*, *Arabidopsis thaliana*, and *Brassica napus*, respectively. Segmental duplication and purifying selection are the causes of the *BolPAL* gene’s amplification and evolution. The *BolPAL* genes with comparable intron–exon architectures and motifs were grouped together in the same clade. Three categories comprised the *cis*-regulatory elements: abiotic stressors, phytohormones, and light. According to the results of the qRT-PCR experiments, the majority of the *BolPAL* genes were expressed highly under MeJA, a low temperature, and a high temperature, and they were downregulated under ABA. Under white light (100 µmol m^−2^ s^−1^) with 50, 100, or 150 µmol m^−2^ s^−1^ far-red (FR), only a small number of the *PAL* genes were expressed at 50 and 100 µmol m^−2^ s^−1^ FR, while the majority of the *PAL* genes were slightly elevated at 150 µmol m^−2^ s^−1^ FR. This work offers a theoretical foundation for molecular breeding research to investigate the role of *BolPAL* genes and their role in anthocyanin biosynthesis.

## 1. Introduction

Phenylalanine ammonia-lyase (PAL) controls the rate at which phenylalanine enters the phenylpropanoid metabolic pathway, which in turn influences the formation of the secondary metabolites in plants, including flavonoids, lignin, and hydroxycinnamic acid amide (HCAA) [1,2]. It is a crucial enzyme that catalyzes the initial stage of the phenylpropanoid pathway and serves as an enzyme to connect the primary and the secondary phenylpropanoid metabolisms [3]. These metabolites are key factors for plant growth, development, and stress tolerance [4]. First discovered in 1961 in barley [3], the *PAL* gene is typically found in plants as a gene family that spans multiple members in various species, including four members in willow [5]. Nevertheless, there is a lack of extensive understanding regarding the complete distribution of the genes related to phenylalanine ammonia-lyase (PAL) throughout the entire genome of *B. oleracea*, a highly nutritious crop.

A study showed that the transcription factors CsbHLHv and CsMYB regulate the *CsPAL* gene, which contributes to anthocyanins synthesis [6]. In a study, it was shown that in the shoots of purple-leaved tea plants, the *CsPAL4* gene expressed and exhibited a significant positive correlation with anthocyanin content [6]. A different study showed that in red and white grapes, *VvPAL1* and *VvPAL5* are involved in the production of anthocyanins [7]. In another study, 14 days after wheat stripe rust struck the studied plants, the plants that had had the *TaPAL32* and *TaPAL42* genes silenced displayed a more severe illness than the control plants [8]. In a different study, the majority of *StPALs* in potatoes responded to drought and high temperatures [9]. In another study, *AtPAL1* and *AtPAL2* were strongly expressed in response to temperature changes and nitrogen stress, which resulted in the formation and accumulation of flavonoids in *A. thaliana* [10]. In a different study, double-knockout mutants of *AtPAL1* and *AtPAL2* resulted in an increased resistance to drought stress, an increased susceptibility to ultraviolet-B (UV-B) radiation, and a decreased biosynthesis of flavonoids and anthocyanins [10]. These studies collectively show that plant tolerance to external stressors is thus significantly influenced by *PAL* genes.

In plants, the PAL proteins are comparatively conserved. Plants differ greatly in the amount of PAL protein they have, but their molecular weight is generally constant, ranging from 275 to 330 kDa [11,12]. The *PAL* family is usually made up of several *PALs*, and each member of the family responds differently to biotic and abiotic stressors and has a unique pattern of expression [13]. The *PAL* gene family has been extensively examined in different crops, including wheat (*Triticum aestivum* L.) [8], walnut (*Juglans regia*) [14], rice (*Oryza sativa*) [15], coleus (*Solenostemon scutellarioides* (L.) Codd) [13], cottonwood (*Populus trichocarpa*) [16], and *A. thaliana* [17]. However, the *PAL* gene family in *B. oleracea* and its expression under various abiotic stressors have not been identified yet.

Renowned for its delicate stems and nourishing leaves, Chinese kale (*B. oleracea*), sometimes referred to as kailaan or gai lan, is a widely used leafy vegetable in Chinese cuisine [18,19]. We performed a genome-wide investigation to identify the *BolPALs* in *B. oleracea*’s genome to evaluate the evolutionary relationships, conserved motifs, gene structure, and *cis*-acting regions in the promoter sequences of *BolPALs* in order to investigate their structural diversity and evolution. Furthermore, we represented the expression profile of *BolPALs* that were subjected to various treatments such as abscisic acid (ABA), methyl jasmonate (MeJA), a low temperature, a high temperature, and light with varying far-red capacities. These results will not only help us better understand how *BolPALs* respond to abiotic stress, but they will also make it easier to study the biological roles of an important gene family and identify possible objectives for productive breeding in *B. oleracea* crops under a variety of abiotic stressors.

## 2. Results

### 2.1. Identification and Characterization of the BolPALs

A total of nine *BolPAL* genes were recognized from the whole genome of *B. oleracea* via the *Brassica* database, http://www.brassicadb.cn/ (accessed on 12 May 2024) [20], as shown in Table 1. The names of these genes are *BolPAL1-1, BolPAL1-2*, *BolPAL2-1*, *BolPAL2-2*, *BolPAL2-3*, *BolPAL2-4*, *BolPAL3-1*, *BolPAL3-2*, and *BolPAL4*. Further, we verified the *PAL* gene-specific lyase aromatic domain (PF00221) in all the identified genes via the NCBI conserved domain database, https://www.ncbi.nlm.nih.gov/Structure/cdd/cdd.shtml (accessed on 12 May 2024) (Figure S1). Detailed information on all the *BolPALs* is given in Table 1. The coding sequences ranged from 801 bp (*BolPAL3-1*) to 2172 bp (*BolPAL2-2* and *BolPAL2-4*), and the protein sequences ranged from 267 aa to 724 aa, while the predicted molecular weights varied from 29.8 to 78.6 MW/kDa, and the isoelectric points varied from 5.54 (*BolPAL2-3*) to 7.71 (*BolPAL3-1*). During subcellular localization prediction, we found that all the BolPAL proteins were located on the cytoplasm and only BolPAL1-1 was found on the plastid. Moreover, 4, 7, and 17 *PAL* genes from *A. thaliana*, *B. rapa*, and *B. napus*, respectively, were also identified (Table S2).

### 2.2. Phylogenetic Analysis of the PAL Family Genes

**Figure 1:**
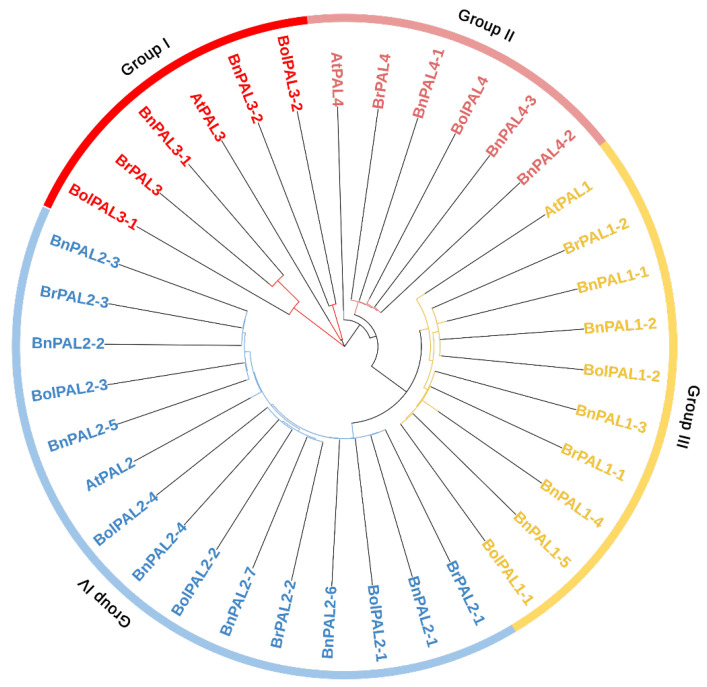
PAL proteins identified from *B. oleracea* (9 BolPALs), *B. napus* (17 BnPALs), *B. rapa* (7 BrPALs), and *A. thaliana* (4 AtPALs) were used to create the neighbor-joining phylogenetic tree. Node distributions divided all the *PAL* genes into four primary groups (I, II, III, and IV) separated by different colors.

Based on the phylogenetic results, all the *PAL* genes from *B. oleracea, B. rapa*, *A. thaliana*, and *B. napus* were separated into four major groups (I, II, III, and IV), based on their node distributions, as shown in Figure 1. Group I contained 2 *BolPAL* (*BolPAL3-1* and *BolPAL3-2*), 1 *AtPAL3*, 2 *BnPAL* (*BnPAL3-1* and *BnPAL3-2*), and 1 *BrPAL* (*BrPAL3*). Group II consisted of 1 *BolPAL* (*BolPAL4*), 1 *AtPAL4*, 3 *BnPAL* (*BnPAL4-1*, *BnPAL4-2*, and *BnPAL4-3*), and 1 *BrPAL* (*BrPAL4*). Group III contained 2 *BolPAL* (*BolPAL1-1* and *BolPAL1-2*), 1 *AtPAL1*, 5 *BnPAL* (*BnPAL1-1* to *BnPAL1-5*), and 2 *BrPAL* (*BrPAL1-1* and *BrPAL1-2*). Group IV consisted of 4 *BolPAL* (*BolPAL2-1* to *BolPAL2-4*), 1 *AtPAL2*, 7 *BnPAL* (*BnPAL2-1* to *BnPAL2-7*), and 3 *BrPAL* (*BrPAL2-1* to *BrPAL2-3*) (Figure 1). The uniqueness of this phylogenetic analysis lies in its thorough comparative methodology, emphasizing specific gene clusters and their preservation across several species. This comprehensive and inclusive study improves the comprehension of *PAL* genes’ evolution, offering a vital structure for future investigations on plant genetics and breeding within the *Brassicaceae* family.

### 2.3. Circos and Synteny Analysis of BolPAL Genes

**Figure 2:**
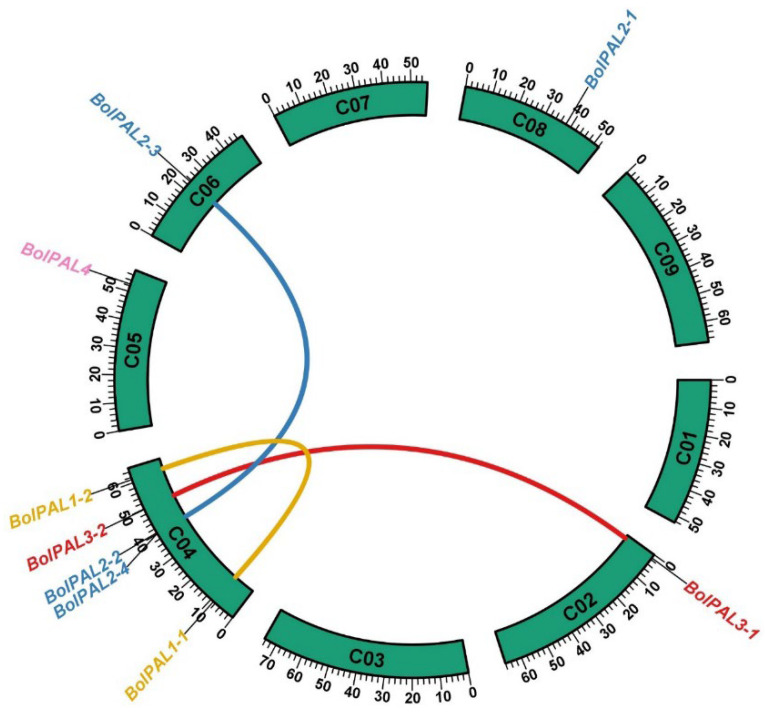
The chromosomal dispersal and inert-chromosomal interaction of the *BolPALs* are illustrated in circular form. Lines of different colors represent the *PAL* genes’ pairs in *B. oleracea*.

The objective of employing a Circos analysis in the investigation of a gene family is to visually represent the genomic distribution and syntenic connections among the genes across various chromosomes and species [21]. This study aids in the identification of gene duplication, conservation, and rearrangement patterns, which offer constructive understandings of the evolutionary dynamics and functional diversity of the *BolPAL*s. Our Circos analysis revealed that 9 *BolPAL* genes are distributed unevenly on 5 out of 10 chromosomes of *B. oleracea* (Figure 2). C04 contains the five (*BolPAL1-1*, *BolPAL1-2*, *BolPAL2-2*, *BolPAL2-4*, and *BolPAL3-2*) *BolPAL* genes, while C02, C05, C06, and C08 contain one gene each (Figure 2). In addition, two paralogous and one homologous gene were identified. Moreover, we found only one tandem and one proximal distribution on chromosome C04. These results indicated that the *BolPAL* gene family has evolved through not only whole genome triplication, but also through tandem and proximal duplications.

**Figure 3:**
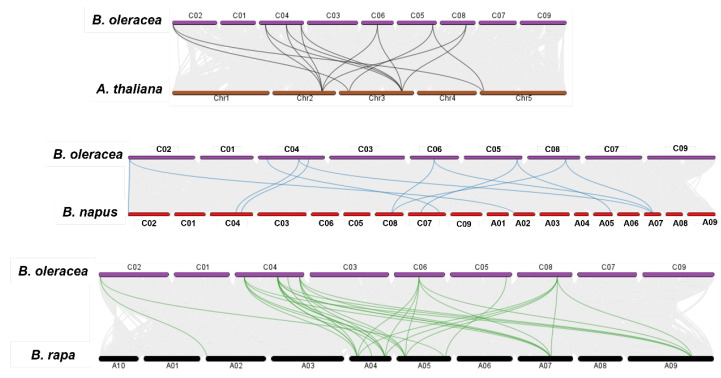
Graphical illustration of syntenic association of *B*. *oleracea* with *A*. *thaliana*, *B*. *rapa*, and *B*. *napus*. Gray lines show all syntenic pairing, while colored lines represent the syntenic association of *PAL* genes between *B. oleracea*, *A. thaliana*, and *B. napus* genomes.

Evolutionary syntenic relations of the *BolPAL* genes with the *A. thaliana*, *B. napus*, and *B. oleracea* genomes were observed via a collinearity analysis (Figure 3). According to the result, all the *BolPAL* genes have syntenic connotations with the *AtPAL*, *BnPAL*, and *BrPAL* genes. In detail, the *AtPALs* from Chr2 are associated to the *BolPALs* from C04, C05, C06 and C08. The *AtPALs* from Chr3 made syntenic associations with the *BolPALs* from C02, C04, C05, and C08. The *AtPALs* from Chr5 are associated to the *BolPALs* from C02 and C05. In the same manner, syntenic associations between the *PAL* genes from *B. oleracea*, *B. rapa*, and *B. napus* are also diverse (Figure 3). These results show that diverse duplication processes in the whole genome contributed to the evolutionary development of the *BolPAL*s.

### 2.4. Gene Structure and Conserved Motif Analysis

**Figure 4:**
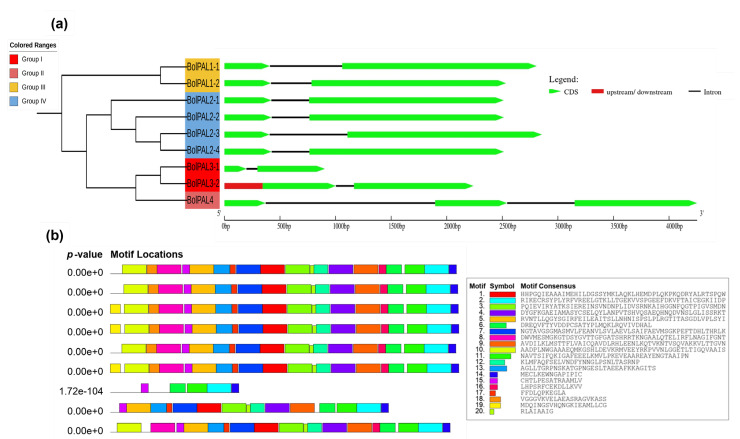
Structural and conserved motif analysis of the *PAL* family from *B. oleracea*. Based on phylogenetic analysis, *PAL* genes were divided into four different groups (I–IV). (**a**) Gene structural analysis. (**b**) The conserved motifs of BolPALs.

An examination of gene structure and conserved motifs was conducted to gain a better insight into the arrangement and functional components of the genes. This analysis provides valuable insights into the evolutionary history and regulatory processes of the genes. To understand the *BolPAL* gene family’s development, the exon–intron configuration and the motif distribution were observed (Figure 4a,b). We observed that all the *BolPAL* genes contain only one intron, except *BolPAL4*, which contains two introns, while two to three exons were identified in the *BolPAL* gene family (Figure 4a). Genes from all the groups were found to have a similar exon–intron distribution. In the same manner, all groups possessed 18–19 motifs distributions in their protein regions except BolPAL3-1 from group I, which contains 5 motifs (Figure 4b). Information of the motifs is given in Table S3. The preserved distribution of introns/exons and motifs within and among several classes of *BolPAL* genes suggests that these genes have retained their structural integrity and functional roles throughout the course of evolution. This evidence indicates that the *BolPAL* genes have a vital and consistent function in growth and regulation.

### 2.5. Recognition of Cis-Acting Elements in the Promoter Region of BolPALs

**Figure 5:**
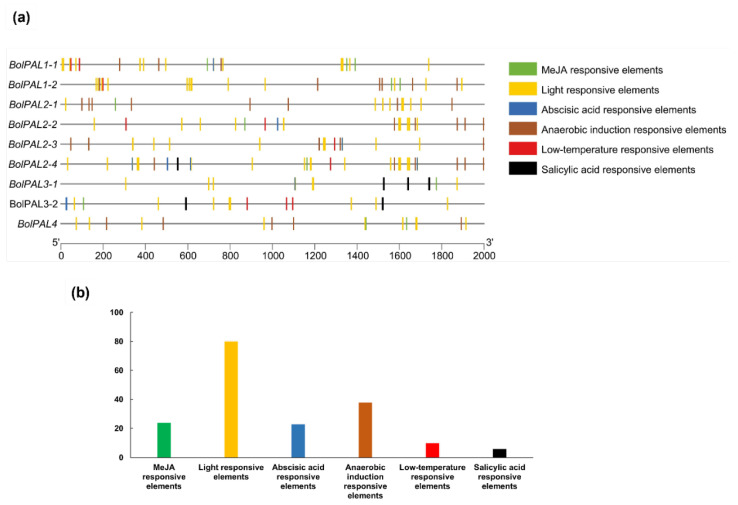
Identification of *cis*-acting elements in the *BolPAL* gene promoters. (**a**) Different abiotic stress (anaerobic, light, and low temperature)- and phytohormone (ABA, MeJA, and SA)-related *cis*-regulatory elements in *BolPALs.* (**b**) Graphical representation of total number of different phytohormones and other abiotic stress-related elements targeting *BolPAL* genes.

The identification of *cis*-acting elements in the promoter region involves the recognition of particular DNA sequences that control the transcription of neighboring genes [22]. These elements are essential for comprehending the regulation of gene expression in response to different physiological and environmental signals [23]. A study of the *cis*-elements present on 2000 base pairs from the upstream region of each *BolPAL* gene was performed to find out the regulatory pathway and the function of genes (Figure 5; Table S4). Three diverse phytohormone-related regulatory elements were identified in the *BolPAL* genes, including ABA (ABRE-ACGTG), MeJA (TGACG-motif), and salicylic acid (SA) (TCA-element) (Figure 5a). Aside from the phytohormones, we also discovered other factors, such as anaerobic condition-responsive (ARE-AAACCA), light-responsive (G-box and GT1-motif), and low-temperature-responsive (LTR-CCGAAA) *cis*-elements, that we expect will further enhance the activity and regulatory pattern of the *BolPAL* genes (Figure 5a; Table S4).

Moreover, we identified the total number of phytohormone-responsive and abiotic-responsive elements (Figure 5b). The results demonstrated that the promoter region of the *BolPAL* genes is targeted by MeJA-, ABA-, light-, and anaerobic-responsive elements. These phytohormone-related and different stress-related elements in the *BolPALs’* promoter regions predict that transcriptional profiling of these genes may vary due to these stresses.

### 2.6. Investigation of miRNAs Directing BolPALs

**Figure 6:**
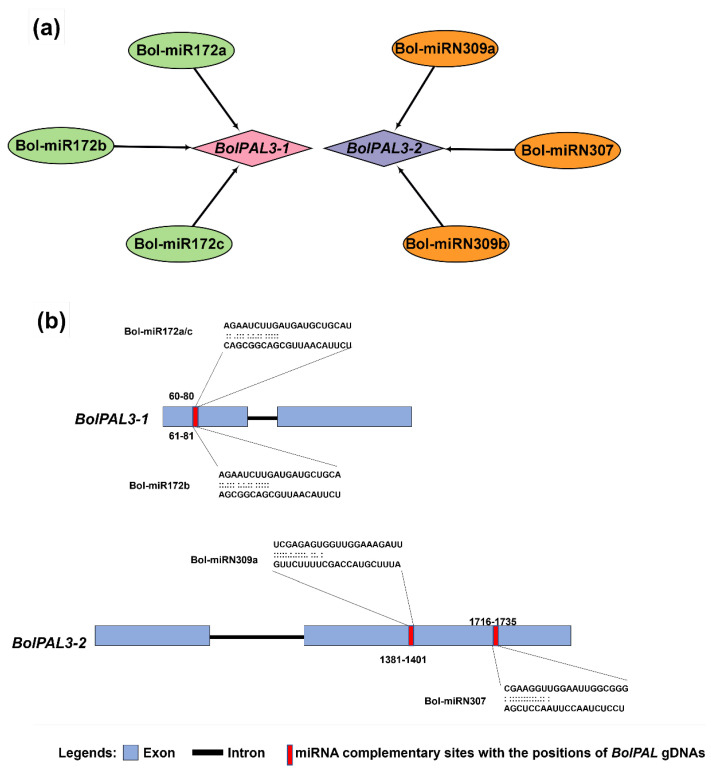
The miRNA targeting sites in the *BolPAL* genes (*BolPAL3-1* and *BolPAL3-2*). (**a**) Network of interaction between BolmiRNAs targeting the *BolPAL* genes while (**b**) showing the targeting sites in *BolPAL3-1* and *BolPAL3-2* genes.

Different biotic stress- and abiotic stress-related responses in many plants were found to have connections with miRNA-directed regulators. Therefore, to enhance our knowledge of the miRNAs controlling the regulation of the *BolPALs*, we identified the miRNAs targeting different *BolPAL* genes, as revealed in Figure 6, in which *BolPAL3-1* and *BolPAL3-2* were found to be targeted by Bol-miR172a-c and BolmiRN309a-c. The conserved miRNA family miR172 is found in many plant species and has been linked to a number of biological processes, such as the regulation of flowering time, the phase transition from vegetative to reproductive growth, and the development of floral organs [24,25,26]. Our findings suggest that the control of *BolPAL3-1* and *BolPAL3-2* by Bol-miR172a-c may have a similar impact on important developmental processes in *B. oleracea*, suggesting a wider functional significance of these miRNAs in the growth and development of plants. Detailed information on these putative miRNAs is presented in Table S5.

### 2.7. GO Annotation of BolPAL Gene Family

To predict the functions of the *BolPALs*, we executed a GO enrichment analysis based on their molecular function (MF), biological process (BP), and cellular component (CC) classes (Figures S2 and S3). These terminologies assist us in predicting the molecular functions of different genes. Complete information on the annotation results is available in Table S6.

The GO-MF enrichment analysis predicted seven enriched terms, ammonia-lyase activity (GO:0016841), catalytic activity (GO:0003824), carbon–oxygen lyase activity (GO:0016835), phenylalanine ammonia-lyase activity (GO:0045548), hydro-lyase activity (GO:0016836), lyase activity (GO:0016829), and transferring alkyl or aryl (other than methyl) groups (GO:0016765). While the GO-BP enrichment analysis found 14 enriched terms, including the L-phenylalanine catabolic process (GO:0006559), the sulfur compound metabolic process (GO:0006790), developmental cell growth (GO:0048588), unidimensional cell growth (GO:0009826), the metabolic process (GO:0008152), cell morphogenesis involved in differentiation (GO:0010770), photoreactive repair (GO:0006281), the alpha-amino acid biosynthetic process (GO:1901607), the developmental process involved in reproduction (GO:0003006), the multicellular organismal process (GO:0032501), fertilization (GO:0007338), localization (GO:0051179), the response to stimulus (GO:0048583), and unidimensional cell growth (GO:0009826).

The GO-CC enrichment annotation examination spotted six enriched terms, cytoplasm (GO:0005737), nucleus (GO:0005634), cellular anatomical entity (GO:0110165), extra cellular space (GO:0005615, cellular anatomical entity (GO:0110165), and protein-containing complex (GO:0032991) (Figure S3). The GO enrichment data suggest that the *PAL* genes have a crucial function in the formation of anthocyanin and the metabolism of phenylpropanoid.

### 2.8. Expression Analysis of BolPAL Genes by Transcriptome Analysis

**Figure 7:**
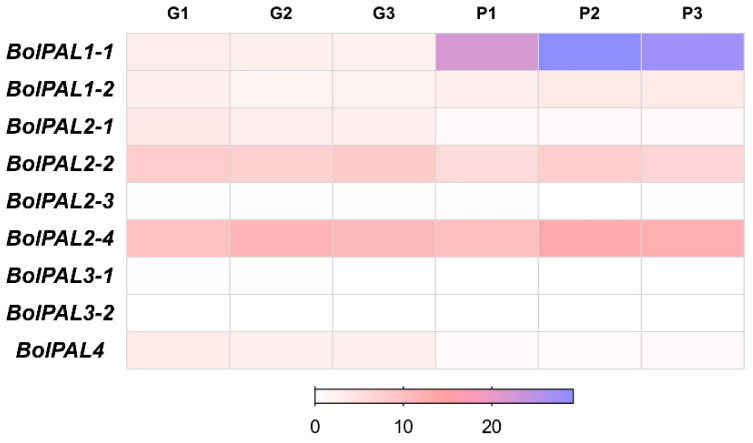
Heatmap of transcriptome expression analysis of *BolPAL* genes in green- (G1, G2, G3) and purple-stalked (P1, P2, P3) Chinese kale (*B. oleracea*).

The increased expression of *PAL* genes can upregulate the production of PAL enzymes, leading to a higher flow of substrates via the phenylpropanoid pathway and finally resulting in greater amounts of anthocyanins [27,28]. In contrast, the reduction or suppression of *PAL* genes might lead to a decrease in anthocyanin synthesis [29,30]. According to the transcriptome analysis [31], out of nine, only one gene, *BolPAL1-1*, was upregulated, while two genes, *BolPAL2-1* and *BolPAL4,* were downregulated in purple-stalked Chinese kale (*B. oleracea*), as compared to green (Figure 7; Table S7). These results suggest that the three *PAL* genes are essential for the creation of anthocyanin and purple color. It has been reported that *PAL* genes catalyze the initial step in the phenylpropanoid pathway, which results in the production of phenolic chemicals [31]. These molecules serve as precursors for the process of anthocyanin biosynthesis, which leads to the build-up of pigments that are responsible for the purple coloration in the plant [31].

Gaining understanding of the function of *PAL* genes in anthocyanin biosynthesis in *B. oleracea* is crucial for plant breeders and biotechnologists who seek to create cultivars with a heightened nutritional value, aesthetic appeal, and potentially greater resilience to stress by augmenting anthocyanin levels. Modifying the expression of the *PAL* gene using genetic engineering or selective breeding techniques could be employed to accomplish these objectives.

### 2.9. Expression Analysis of BolPAL Genes under Phytohormone, Abiotic, and Light Treatments

**Figure 8:**
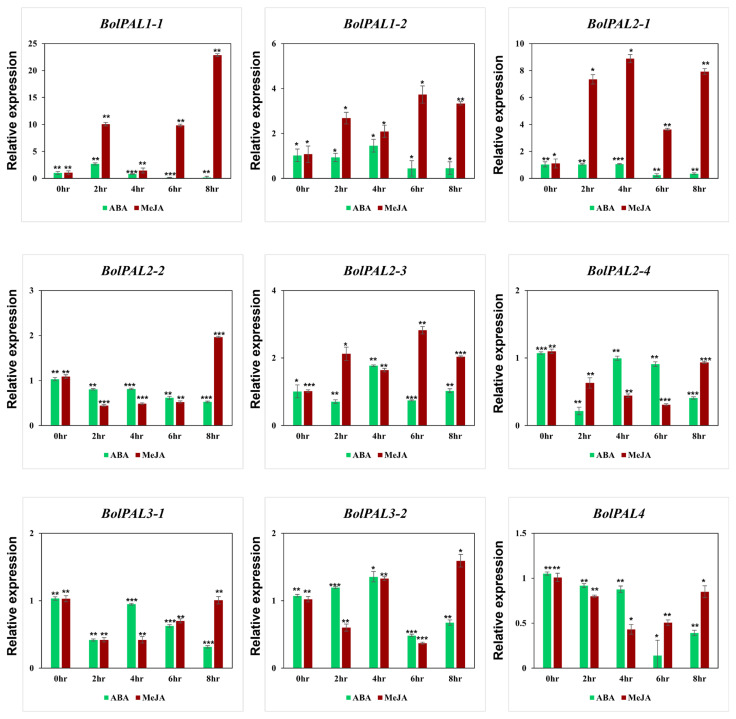
Graphical demonstration of the expression profile of the *BolPAL* genes in *B. oleracea* under phytohormonal stresses (ABA and MeJA) (0 h as control, 2 h, 4 h, 6 h, and 8 h). The 2^−∆∆CT^ method was used to examine the results. (*, *p* < 0.01; **, *p* < 0.005; ***, *p* < 0.001).

Under different phytohormones, the growth of plants can be affected at physiological, molecular, and biochemical levels. Our *cis*-element identification revealed the presence of ABA-, MeJA-, and SA-responsive elements in the *BolPAL* genes. Therefore, we applied ABA and MeJA phytohormones to observe their role in the expression of the *BolPAL* genes (Figure 8). Six members of this gene family were expressed under MeJA treatment at different times, which include *BolPAL1-1* at 2 h, 6 h, and 8 h; *BolPAL1-2* and *BolPAL2-1* at 2 h, 4 h, 6 h, and 8 h; *BolPAL2-2* at 8 h; *BolPAL2*-*3* at 2 h, 6 h, and 8 h; and *BolPAL3*-*2* at 8 h, while the remaining three genes were found to be downregulated (Figure 8). Under ABA treatment, almost all the members of this gene family were found to be downregulated, except for a few genes that were partially expressed at different time points, such as *BolPAL1-2* and *BolPAL2-3* at 4 h, and *BolPAL3-2* at 2 h and 4 h (Figure 8). Three genes, named *BolPAL1-2*, *BolPAL2-3*, and *BolPAL3-2,* are common genes that displayed elevated expression under both the phytohormones (MeJA and ABA), which predicts that these three genes are the most important for further studies and can be helpful in future breeding programs of *B. oleracea* crops.

**Figure 9:**
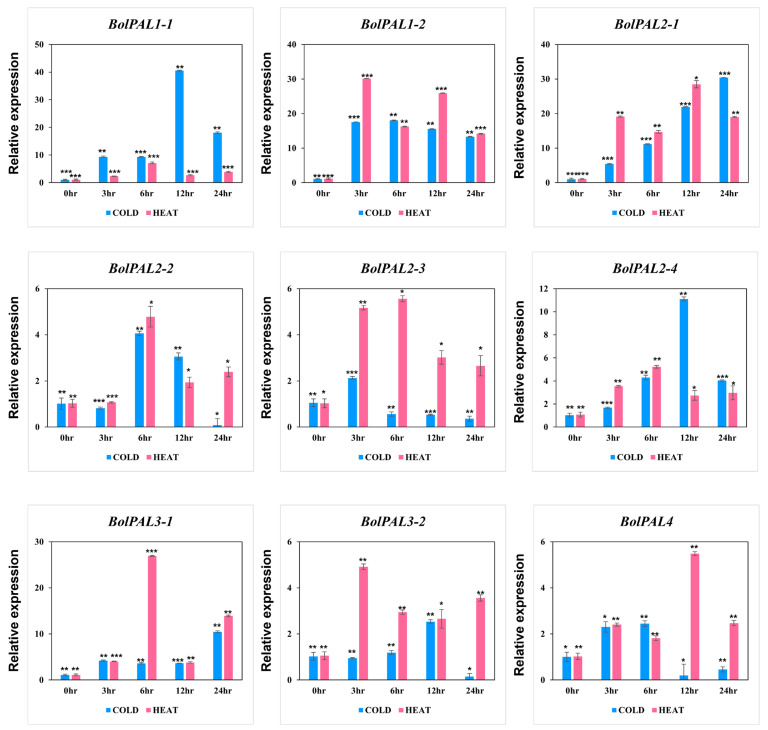
Graphical presentation of *BolPAL* genes under low and high temperature stresses at different time points (0 h as control, 2 h, 4 h, 6 h, and 8 h). The 2^−∆∆CT^ method was used to examine the results. (*, *p* < 0.01; **, *p* < 0.005; ***, *p* < 0.001).

Surprisingly, all the *BolPAL* genes were regulated under cold and heat stress at different time points (Figure 9). In detail, under cold stress, *BolPAL1-1*, *BolPAL2*-*4*, *BolPAL3*-*2* at 12 h, *BolPAL1*-*2, BolPAL2*-*2,* and *BolPAL4* at 6 h; *BolPAL2*-*1* and *BolPAL3*-*1* at 24 h; and *BolPAL2*-*3* at 3 h expressed strongly. Under heat stress, five genes, including *BolPAL1*-*1*, *BolPAL2*-*2*, *BolPAL2*-*3*, *BolPAL2*-*4*, and *BolPAL3*-*1* expressed at 6 h; *BolPAL1*-2 and *BolPAL3*-*2* at 3 h; and *BolPAL2*-*1* and *BolPAL4* at 12 h showed elevated expressions. Significant changes in the expression of the *PAL* genes could result in the promoted synthesis of phenolic compounds, which function as antioxidants and enhance resistance to low and high temperatures [32,33]. These results indicate that *PAL* genes are crucial in response to studied stress conditions, especially temperature-related stresses.

**Figure 10:**
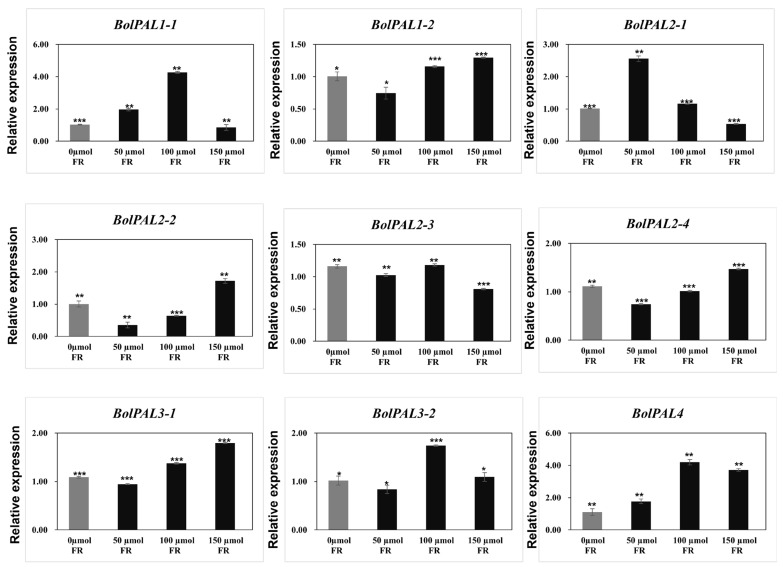
Graphical illustration of *BolPAL* genes under white light (100 µmol m^−2^ s^−1^), plus 50, 100, and 150 µmol m^−2^ s^−1^ far-red (FR). The 2^−∆∆CT^ method was used to examine the results. (*, *p* < 0.01; **, *p* < 0.005; ***, *p* < 0.001).

For light stress, white light with a 100 µmol m^−2^ s^−1^ intensity as a control, plus 50, 100, and 150 µmol m^−2^ s^−1^ FR (FR) were used as stresses. Four genes (*BolPAL1*-*2*, *BolPAL2*-*2*, *BolPAL2*-*4*, and *BolPAL3*-*1*) were upregulated at 150 µmol m^−2^ s^−1^ FR, whereas *BolPAL1*-*1*, *BolPAL3*-*2*, and *BolPAL4* were expressed at 100 µmol m-2 s-1 FR, and only *BolPAL2*-*1* was upregulated at 50 µmol m^−2^ s^−1^ FR (Figure 10). Far-red (FR) light stress activates phytochrome signaling, triggering hormonal pathways and transcription factors that upregulate gene expression [34]. This increases phenolic compound production, helping plants manage oxidative stress and reinforce structural defenses [35,36]. Our results indicate that *BolPAL* genes play a major role in stress responses and adaptation, as evidenced by the increased expression of these genes under MeJA, ABA, heat, cold, and light stresses. These outcomes can be helpful in developing new cultivars that are more resilient and are stress-tolerant. Additionally, understanding the regulation of *BolPAL* genes under various stress conditions might aid future studies that aim to enhance agricultural performance and stability in dynamic environmental changes.

## 3. Discussion

Plants use the phenylpropanoid pathway as a major metabolic process to produce a wide range of secondary metabolites, including phenolic acids, lignin, and flavonoids [27]. The enzyme phenylalanine ammonia-lyase (PAL) catalyzes the deamination of the amino acid phenylalanine, which is the first step in this route [31,37]. Phenylalanine is transformed by PAL into trans-cinnamic acid, which thereafter proceeds via a sequence of enzymatic processes to yield different phenylpropanoids [27]. These substances are essential for UV protection, pigmentation, structural integrity, and pathogen defense systems in plants [27,38]. Specifically, plants get their red, blue, and purple hues from anthocyanins, which are generated from flavonoids [31,39,40]. Because they catalyze the first and rate-limiting step in this route, the *PAL* genes are essential for controlling the flow of metabolites into the phenylpropanoid biosynthesis [27]. Plant growth, stress responses, and general adaptability are all impacted by the activity and expression levels of the *PAL* genes, which in turn directly affect the synthesis of phenolic chemicals [41,42].

The PAL enzyme plays a crucial role in the secondary phenylpropanoid pathway, enabling the production of lignin, which provides mechanical support, the synthesis of pigments like anthocyanins and flavonoid nodule factors, and defense against biotic and abiotic stresses [43,44]. Thus, a great deal of research has gone into figuring out the signal transduction pathways connected to these mechanisms. As an example, in *A. thaliana*, overexpressed *GmPAL1.1* improved seed maturity under stress conditions of high temperatures and humidity [45]. *PtPAL1* expression in poplar was upregulated in response to leaf damage [11]. Furthermore, pepper plants that had *CaPAL1* silenced were more vulnerable to Xcv susceptibility [11]. Quadruple mutants of all thte *AtPAL* genes (*AtPAL1*-*AtPAL4*) also showed abnormalities and a significant decrease in the quantities of stored salicylic acid [17].

In this study, we used the *Brassica* database to identify nine *BolPAL* genes that aligned with the *PAL* genes found in other plants, such as cucumber [46], hickory nut [47], and tea tree [6]. These *BolPAL* genes are notable for having conserved motifs and similar gene structures. These conserved motifs may govern molecular processes in PAL genes in several plant species [11]. The *PAL* genes from *B. oleracea*, *A. thaliana*, *B. rapa,* and *B. napus* were analyzed using a phylogenetic analysis, which showed that the *BolPAL* showed strong affinities with *A. thaliana* and two additional *Brassica* crops, *B. napus* and *B. oleracea*. The *BolPALs* exhibited a high degree of genetic variety, which may be explained by the genome-wide triploidization processes that occurred in *Brassica* after their separation from the *A. thaliana* lineage [48,49]. The *BolPAL* family members are unevenly distributed among five chromosomes in *B. oleracea*, which is comparable to the *PAL* genes, which are dispersed on a few chromosomes in other species [11]. It has been found that, during the evolution of plants, procreation and ancestor modification can produce many family members, which can be grouped together on a single or multiple chromosomes [8,50], indicating that the *BolPAL* family’s growth is being driven by chromosomal duplication, while the diverse range of developments among *Brassica* species may be explained by differences in the gene families’ sizes [51]. Furthermore, it is possible that functional redundancy among close relatives can be quantified, which may lead to the development of more potent methods for detecting traits that are deficient in loss-of-function research involving *B. oleracea* breeding.

The growth and activation of the *PAL* gene’s expression are also governed by the promoter activity of the gene, and the promoter region often includes a range of *cis*-regulatory elements. The *cis*-acting components of the *BolPAL* promoter were predicted and analyzed, and the results suggest that the *BolPALs* may react to light, abiotic stress, and several phytohormones. Based on phytohormone, abiotic, and light treatments, five members of the *BolPAL* gene family (*BolPAL1-1*, *BolPAL1-2*, *BolPAL2-1*, *BolPAL2-2*, and *BolPAL3-2*) were found to have high expression at various times under MeJA treatment (Figure 8). Almost all the members of this gene family were shown to be suppressed when treated with ABA, with the exception of a few genes, such as *BolPAL1-2*, *BolPAL2-3*, and *BolPAL3-2*, which merely expressed highly at different times (Figure 8). Interestingly, at different times, all the *BolPAL* genes were substantially regulated under heat and cold stresses (Figure 9). White light with 100 µmol m^−2^ s^−1^ intensity, plus 50, 100, and 150 µmol m^−2^ s^−1^ far-red (FR), were utilized as stressors to further investigate the possible role of the *BolPAL* genes. At 150 µmol m^−2^ s^−1^ FR, the majority of the genes were slightly elevated; however, only a small number of genes were expressed at 50 and 100 umol m^−2^ s^−1^ FR (Figure 10). These findings suggest that the *PAL* genes are essential for adapting to the stressors being studied, especially temperature-related stress.

Similar findings were also discovered in studies conducted on other species [17,52,53,54]. The elevation of the *BolPAL* expression levels at low temperatures in this study is consistent with the upregulation of *CsPAL* gene expression levels during cold stress [46]. After receiving exogenous ABA treatment, *CsPAL2* and *SlPAL5*s’ expression was dropped [55]; in the same study, *BolPAL’s* expression was likewise downregulated; following MeJA treatment, six potato *PAL* genes were considerably upregulated and four were dramatically downregulated [9]. In this study, under MeJA treatment, four *BolPAL* genes dramatically downregulated, whereas five of them showed considerable upregulation. *BrPAL* genes were downregulated under 60 umol m^−2^ s^−1^ FR [52]. In our results, only *BolPAL1-1* and *BolPAL2-1* were slightly upregulated under 50 umol m^−2^ s^−1^ FR. In conclusion, under a variety of abiotic stressors and phytohormone treatments, the expression of most *BolPAL* genes were regulated at different time points. Members of the *BolPAL* gene family can be differentially induced to express themselves, depending on when stress and phytohormone treatments occur. These variations in gene expression can be beneficial to plants as they may enable them to have varying degrees of stress resistance and play a significant role in hormone signaling.

## 4. Materials and Methods

### 4.1. Identification of BolPALs in B. oleracea

The *Brassica* database (BRAD) http://www.brassicadb.cn/ (accessed on 12 May 2024) was used to identify and obtain all the sequences of the *BolPALs* [20], while the protein sequences of *A. thaliana* were downloaded from the *Arabidopsis* Information Resource (TAIR) at http://www.arabidopsis.org/ (accessed on 12 May 2024) [56]. To verify all the *PAL* genes of *B. oleracea*, we performed an analysis to find the *PAL* gene-specific lyase aromatic domain (PF00221) in all the protein sequences by using the NCBI conserved domain database, https://www.ncbi.nlm.nih.gov/Structure/cdd/wrpsb.cgi/ (accessed on 12 May 2024), followed by the TBtools software V 1.098: https://github.com/CJ-Chen/TBtools (accessed on 12 May 2024) [57]. An online tool, ExPASy (http://web.expasy.org/protparam/) (accessed on 12 May 2024), was employed to measure the physiochemical properties (molecular weight and isoelectric points) of the *BolPALs*. The gene structure of the *BolPALs* was identified and constructed by accessing the Gene Structure Display Server 2.0, http://gsds.gao-lab.org/ (accessed on 12 May 2024), while a conserved motif analysis of the BolPAL proteins was carried out using MEME (V 4.11.4; accessed on 12 May 2024).

### 4.2. Phylogenetic Tree and Synteny Investigation of BolPALs

To observe their evolutionary relationships, the protein sequences of the *PAL* family from *B. oleracea, B. rapa*, *A. thaliana*, and *B. napus* were used. The neighbor-joining (NJ) method, with 1000 bootstrap replicates, was used to align and construct the peptide sequences using MEGA-X (accessed on 12 May 2024). Collinearity and synteny analysis of all *PAL* genes were performed through TBtools software V 1.098; https://github.com/CJ-Chen/TBtools/ (accessed on 12 May 2024) [57].

### 4.3. Cis-Elements Investigation in the Promoter Region of BolPALs

To detect *cis*-regulatory elements, two thousand base pairs from upstream of the genome sequences of each *BolPAL* were taken from BRAD, http://www.brassicadb.cn/ (accessed on 12 May 2024) [20]. An online tool called PlantCARE, http://bioinformatics.psb.ugent.be/webtools/plantcare/html/ (accessed on 12 May 2024), was accessed to analyze the promoter regions and the prediction of *cis*-regulatory elements. The obtained results were presented using TBtools software V 1.098, https://github.com/CJ-Chen/TBtools/ (accessed on 12 May 2024) [57].

### 4.4. Prediction of Putative miRNA Targeting BolPALs

The genomic sequences of the *BolPALs* were compiled to categorize possible miRNAs by the psRNATarget database, http://plantgrn.noble.org/psRNATarget/ (accessed on 12 May 2024). The Cytoscape V3.8.2, https://cytoscape.org/download.html/ (accessed on 12 May 2024), software program was used to construct the network among the predicted miRNAs and the corresponding target *BolPAL*s [58].

### 4.5. Expression Profiling of BolPALs by Transcriptome Data

We used RNA-seq data [31] to highlight the *BolPAL* genes’ expression in green-stalked (ZSJL) and purple-stalked (HJJL, R) Chinese kale (*B. oleracea*). FPKMs (fragments per kilobase of exon model per million mapped reads) were used to analyze the expression values. The heatmap of the *BolPAL* genes’ expression was created using GraphPad Prism 9.0.0 software, accessible at https://www.graphpad.com/, (accessed on 12 May 2024).

### 4.6. Plant Materials and Stress Conditions

In this study, green-stalked (ZSJL), wild-type Chinese kale (*B. oleracea*) that was obtained from the Vegetable Research Institute, Guangdong Academy of Agricultural Sciences, was used for different stress treatments. The vigorous seeds that had a 100% germination rate were selected and sterilized with a 10% hypochlorous acid solution for 5 min. Cycling conditions of 25 °C and 16 h/8 h light/dark were used to grow seeds in the growth chamber. To determine the effect of the phytohormones, three-week-old seedlings were treated with 100 µM abscisic acid (ABA) and 100 µM MeJA by exogenously spraying [59]. All the leaves were collected at 0 h as the control, as well as 2 h, 4 h, 6 h, and 8 h after the treatment was applied. For light stress, white LED light with 100 µmol m^−2^ s^−1^ intensity as the control and white light plus 50, 100, and 150 µmol m^−2^ s^−1^ far-red light (FR) were applied for three weeks, and all the leaves were collected [52]. To analyze the temperature stress, 4 °C for cold stress and 38 °C for heat stress were applied to the three-week-old seedlings under 16 h light/8 h dark conditions, and all the leaves were collected at 0 h as the control, as well as 3 h, 6 h, 12 h, and 24 h after the treatment. Three biological replicates of each treatment were taken and rapidly frozen in liquid nitrogen and stored at −80 °C.

### 4.7. RNA Extraction and qRT-PCR Analysis

The total RNA from the leaf was isolated using the Tiangen RNA Extraction Kit (Beijing, China). The Agilent Bioanalyzer 2100 system confirmed the RNA quantity (Agilent Technologies, Beijing, China). In detail, the leaf samples were ground into a fine powder using a mortar and pestle, followed by adding the lysis buffer, ensured thorough homogenization. Further, ethanol was added to facilitate the RNA binding to the column, and the sample was then transferred to the spin column, then it was centrifuged to bind the RNA to the membrane, then the column was washed with wash buffers to remove contaminants, then the purified RNA was eluted in RNase-free water by centrifugation. The cDNA was synthesized by a cDNA Synthesis SuperMix kit (TransGen Biotech, Beijing, China) and then diluted 10× by adding distilled deionized water. The qRT-PCR was performed with a CFX Connect Real-Time System (Bio-Rad, Hercules, CA, USA) using a SYBER^®^ Green Supermix (Vazyme, Nanjing, China). *BolActin* primers were used as a control to analyze the results. The qRT-PCR reaction was performed as follows: 94 °C for 10 min, followed by 40 cycles of 94 °C for 15 s, 60 °C for 30 s, and 72 °C for 10 s. Three biological replicates were used for each reaction, and then all the data were analyzed using the 2^−∆∆CT^ method. Table S1 contains information on all the primers for the *BolPAL* genes.

## 5. Conclusions

In this study, we identified nine *BolPAL* members that play a crucial role in the metabolic pathway of anthocyanin biosynthesis, located on five different chromosomes. Through a comprehensive and systematic analysis, including conserved structural domains, chromosomal positioning, phylogenetic relationships, and gene expression analyses under various stresses, we observed a strong correlation between the promoter *cis*-elements of the *BolPAL* genes and their expression in response to abiotic stresses, light, and plant hormones. These findings offer novel insights into the functional role of *BolPALs* and contribute to the ongoing development of the regulatory network governing anthocyanin biosynthesis in *B. oleracea*.

## Figures and Tables

**Table 1:** Detailed information of PAL genes in *B. oleracea*.

Gene ID	Gene Name	Start, Stop	Strand	CDS/bp	Protein/aa	MW/kDa	pI	GRAVY	Predicted Sub-Cellular Localization
BolC04g011300.2J	*BolPAL1-1*	8851040, 8853849	+	2157	719	78.09	5.86	−0.144	Plastid
BolC04g062040.2J	*BolPAL1-2*	61207976, 61210509	−	2166	722	78.3	5.97	−0.161	Chloroplast
BolC08g037230.2J	*BolPAL2-1*	38002613, 38005124	−	2169	723	78.6	5.97	−0.169	Cytoplasm
BolC04g039290.2J	*BolPAL2-2*	40458443, 40460957	+	2172	724	78.6	6.03	−0.157	Cytoplasm
BolC06g020590.2J	*BolPAL2-3*	23428035, 23430891	−	2154	718	78.03	5.54	−0.202	Chloroplast
BolC04g038920.2J	*BolPAL2-4*	40141340, 40143854	+	2172	724	78.6	6.03	−0.157	Chloroplast
BolC02g001850.2J	*BolPAL3-1*	1348328, 1349230	+	801	267	29.8	7.71	−0.37	Chloroplast
BolC04g048500.2J	*BolPAL3-2*	49763466, 49765706	−	1734	578	63.98	5.73	−0.146	Cytoplasm
BolC05g056320.2J	*BolPAL4*	52636659, 52640911	−	2118	706	76.23	5.73	−0.133	Cytoplasm

## Data Availability

The data presented in this study are available on request from the corresponding author.

## Supplementary Materials

The following supporting information can be downloaded at: https://www.mdpi.com/article/10.3390/ijms251910276/s1.
