# Supplementary figures and images for: Comprehensive, Genome-Wide Identification and Expression Analyses of Phenylalanine Ammonia-Lyase Family under Abiotic Stresses in Brassica oleracea
Source: Int J Mol Sci. 2024 Sep 24;25(19):10276. doi: 10.3390/ijms251910276 (PMC11476911; doi:10.3390/ijms251910276)

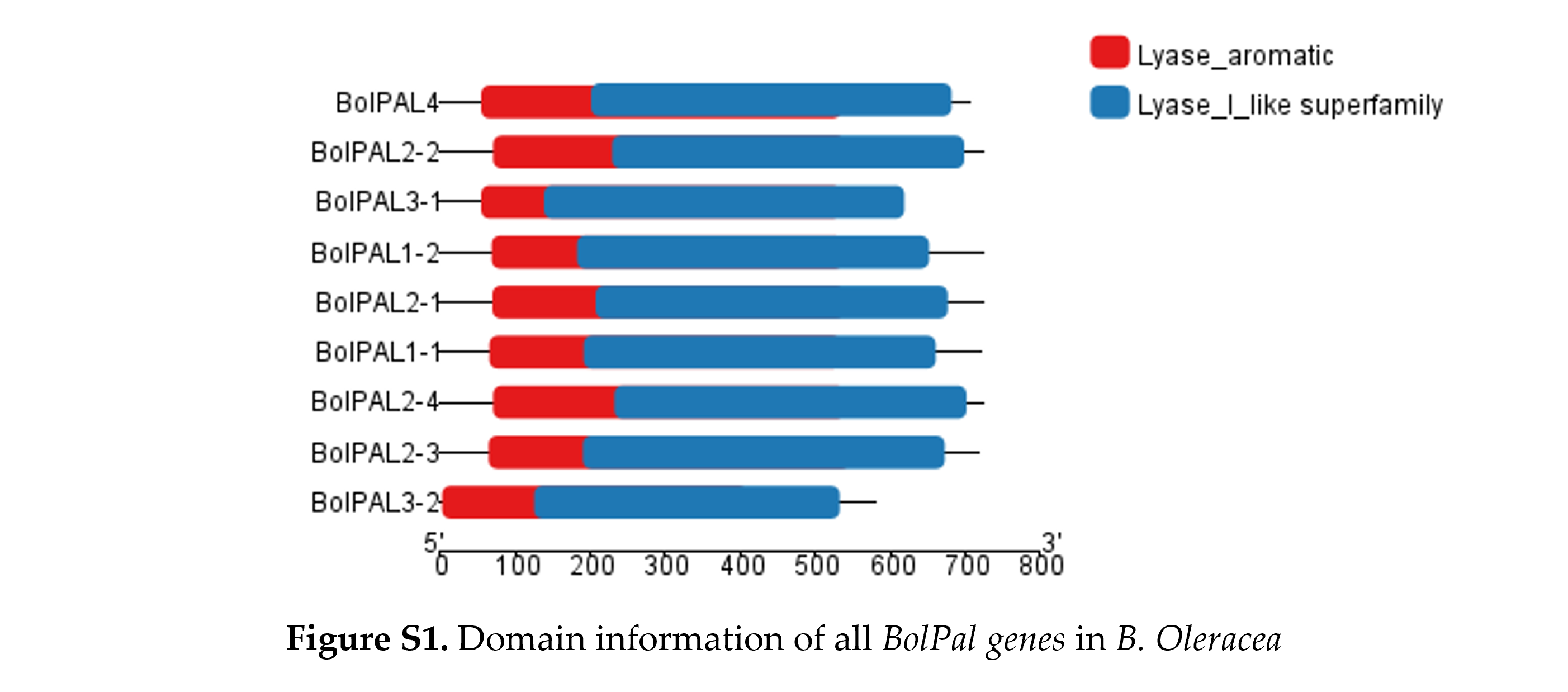

Supplement: Supplementary file 1 [file ijms-25-10276-s001.zip › Supplementary material/Figure S1.png]

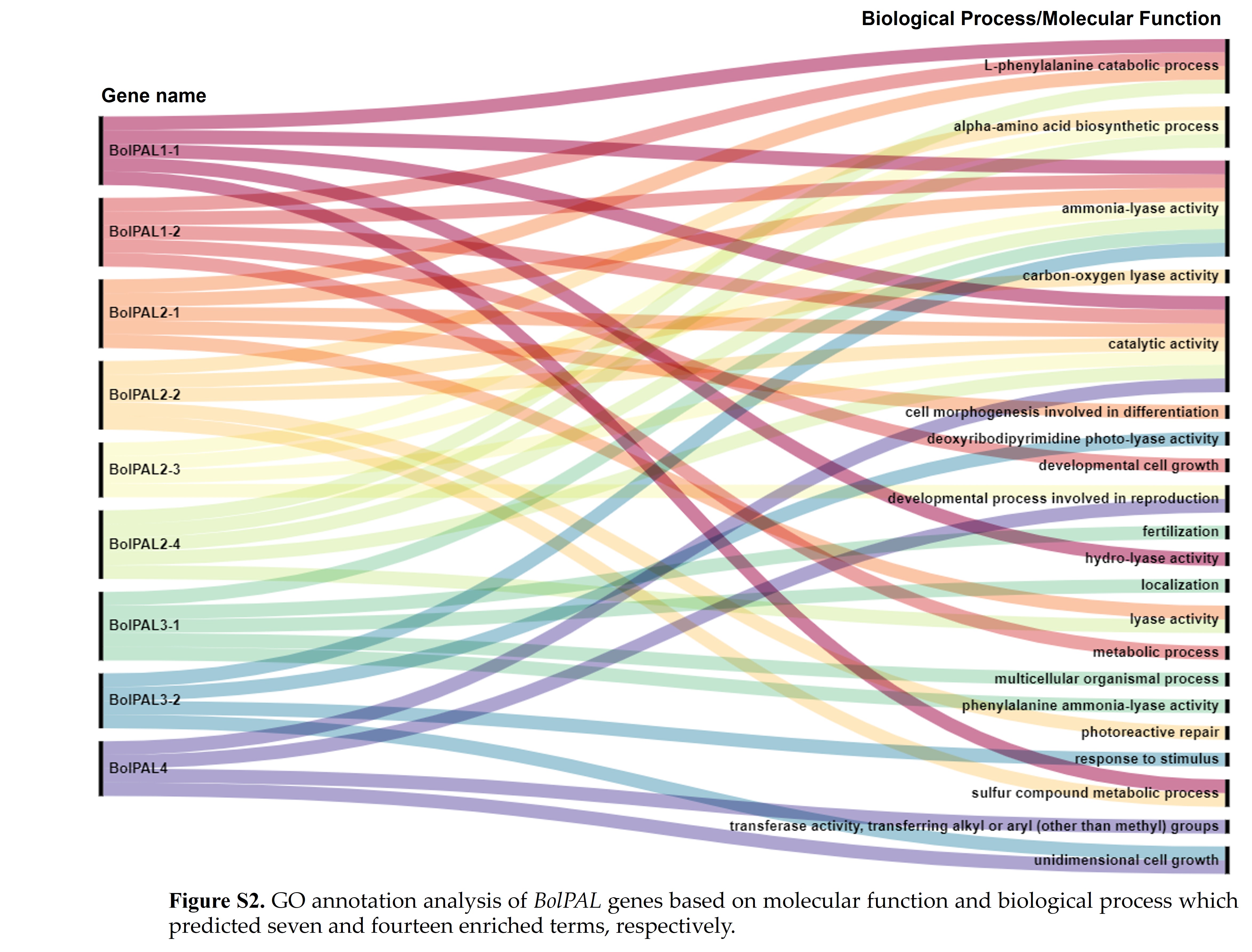

Supplement: Supplementary file 1 [file ijms-25-10276-s001.zip › Supplementary material/Figure S2.png.jpg]

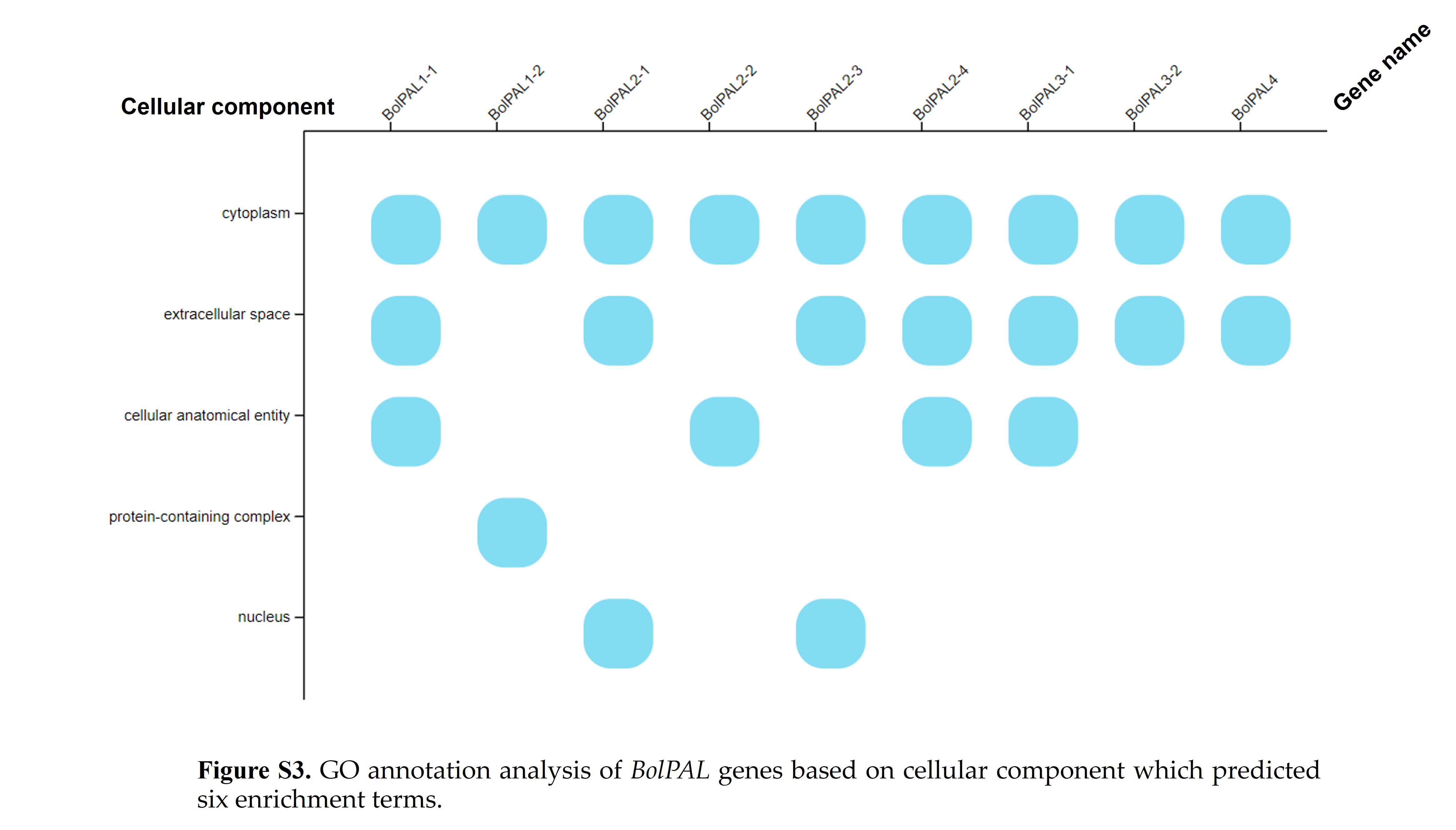

Supplement: Supplementary file 1 [file ijms-25-10276-s001.zip › Supplementary material/Figure S3.png.jpg]
